# Comparison of body composition parameters in the study of the association between body composition and pulmonary function

**DOI:** 10.1186/s12890-021-01543-1

**Published:** 2021-05-25

**Authors:** Caren Ishikawa, Marco Antonio Barbieri, Heloisa Bettiol, Gabriel Bazo, Alexandre A. Ferraro, Elcio Oliveira Vianna

**Affiliations:** 1 Department of Pediatrics, Medical School of Ribeirão Preto, University of São Paulo, Ribeirão Preto, Brazil; 2 Department of Pediatrics, University of São Paulo Medical School, São Paulo, Brazil; 3 Pulmonary Division, Department of Medicine, Medical School of Ribeirão Preto, University of São Paulo, Av. Bandeirantes, 3900, Ribeirão Preto, SP 14048-900 Brazil

**Keywords:** Obesity, Body composition, Respiratory function tests, Epidemiology, Nutrition disorders

## Abstract

**Background:**

The excess adiposity, even in the absence of diseases, is responsible for a decline in pulmonary function, which is considered a predictor of mortality and a risk factor for diseases in several epidemiological studies. However, studies on the association between obesity and pulmonary function have found only few associations or inconclusive results. The aim of the study is to evaluate the association between body composition and spirometric parameters, comparing simple obesity measures such as body mass index (BMI) and waist circumference with more precise body composition measurements such as dual-energy X-ray absorptiometry (DXA) and air-displacement plethysmography (BOD POD).

**Methods:**

This is an observational, cross-sectional study that used data from the 1978/79 Ribeirão Preto birth cohort (São Paulo, Brazil). The study included 1746 participants from the 5th follow-up of the cohort. Linear regressions were calculated to evaluate the association between BMI, waist circumference, waist–height ratio (WHtR), BOD POD- and DXA-measured fat mass percentage, and spirometric parameters FEV1, and FVC.

**Results:**

For every 1-kg/m^2^ BMI increase, FVC decreased by 13 ml in males and by 6 ml in females and FEV1 decreased by 11 ml and 5 ml, respectively. Regarding body composition measurements, for a 1% increase in fat mass assessed by BOD POD, FVC decreased by 16 ml in males and by 8 ml in females and FEV1 decreased by 13 ml and 7 ml, respectively. Hence, negative associations between body measurements and FEV1 and FVC were observed in both genders, especially when using the fat mass measurement and were more expressive in men.

**Conclusion:**

The anthropometric and body composition parameters were negatively associated with the spirometric variables FVC and FEV1. We have also observed that simple measures such as waist-height ratio were sufficient to detect the association of body composition with pulmonary function reduction.

**Supplementary Information:**

The online version contains supplementary material available at 10.1186/s12890-021-01543-1.

## Background

According to the World Health Organization (WHO), the worldwide prevalence of obesity has almost tripled in the last 40 years. In 2016, more than 1.9 billion adults were overweight, corresponding to 39% of the world population, and there were 650 million obese individuals, i.e., 13% of the world population [1]. Obesity is considered the main risk factor for respiratory, metabolic, and cardiovascular diseases. In addition, obesity can alter pulmonary function regardless of the presence of respiratory disease [2–6].

Excess adiposity is known to interfere with the respiratory system through different mechanisms, including altered respiratory mechanics and pulmonary inflammation. Fat accumulation both in the cavities and in the thoracic and abdominal walls hinders the movement of the respiratory muscles, increases pleural pressure and reduces lung compliance, with a consequent decrease in the compliance of the respiratory system as a whole [7−11]. Furthermore, recent studies have demonstrated the influence of adipose tissue on the secretion of proinflammatory cytokines and on changes in the pulmonary immune system [12−16]. These effects are reflected in altered pulmonary function tests, including a reduction in functional residual capacity, expiratory reserve volume, forced expiratory volume in one second (FEV1), and forced vital capacity (FVC) [17, 18].

However, some studies found only a weak association or conflicting results regarding the relationship between obesity and pulmonary function in men and women, mainly because of methodological differences such as the choice of parameter to define obesity [6, 18–21]. Furthermore, new body composition measurement methods have emerged in recent years, which provided new data for the study of the association between body composition and pulmonary function. These methods range from simple measures such as bioimpedance to more complex tests such as magnetic resonance imaging, computed tomography, dual-energy X-ray absorptiometry (DXA), and air-displacement plethysmography (BOD POD). Although they have shown a stronger association between visceral or central fat and pulmonary function, such studies are still scarce. Most of them used small samples [22] or samples restricted to healthy volunteers [19, 23], making generalization difficult, or they did not control for variables that could interfere with pulmonary function and body composition such as exercise and the presence of comorbidities [24, 25].

We hypothesized that the relationship between body composition and pulmonary function reduction may depend on the parameter employed to characterize body composition. The present study aimed to evaluate different body composition parameters and their associations with spirometric values.

## Methods

### Participants

This is a cross-sectional study that used data from a birth cohort started in 1978 in the city of Ribeirão Preto, located 320 km from the capital of São Paulo (SP), Brazil. The estimated population at the time of the study was 682,302 inhabitants, with a Human Development Index of 0.800. Ribeirão Preto is a reference in retail and service activities and is one of the main biomedical research centers in the country [26].

This Brazilian birth cohort study took place between June 1978 and May 1979 in the city of Ribeirão Preto. Its initial objective was to analyze the behavior of some health indicators at birth and their associations with maternal conditions. The second phase of the study focused on the growth of the children (weight and height) during the school years and comprised the period between 1987 and 1989. In a third smaller phase conducted in 1996/97 which only involved men during recruitment for military service, the focus was to study life and health conditions at 18 years of age. The fourth phase of the study was conducted between 2002 and 2004 and was aimed at determining the association between events that had occurred from the prenatal period until the beginning of adult life [27].

In 2016, the data collection for the study “Determinants throughout the life cycle of obesity, precursors of chronic diseases, human capital and mental health” was started, which corresponds to the fifth follow-up of the cohort. The main objective of this phase was to investigate precursors of adult chronic non-communicable diseases, body composition and essential health aspects such as human capital, mental health, and other disorders. The priority was to contact subjects who had participated in the previous phase in 2002 (n = 2103), with 1117 (53.1%) subjects participating in the current data collection. There were also 658 subjects of the original cohort who had not participated in the 2002 follow-up, totaling 1775 subjects.

**Fig. 1:**
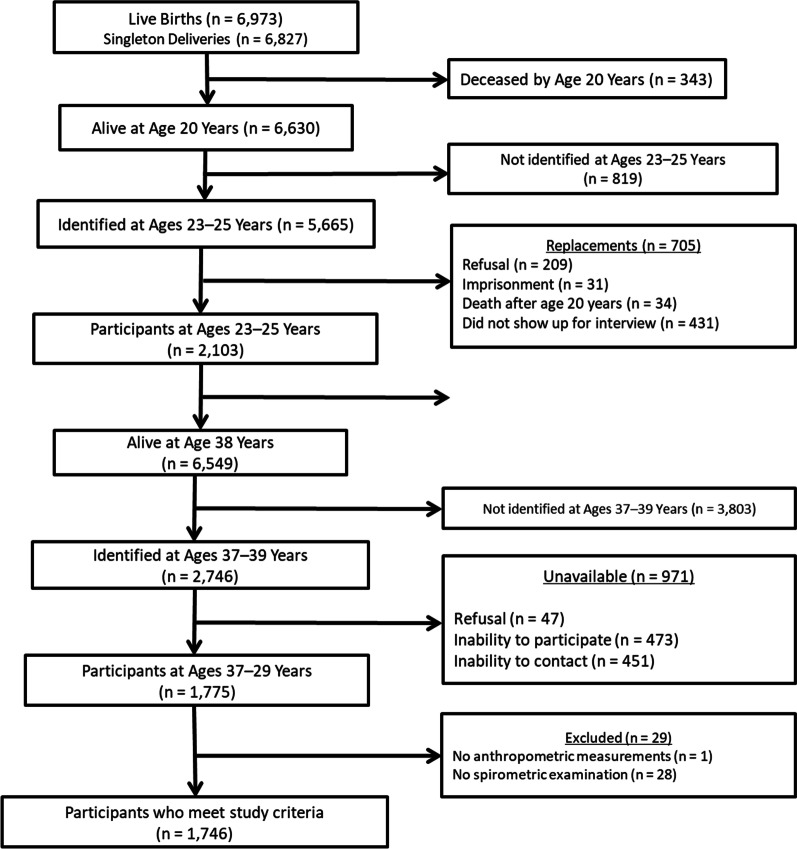
Legend. Sample of the study derived from the birth cohort started in 1978

The present study used data collected during the fifth phase of the cohort. All participants who underwent spirometry (n = 1775) were included. Participants without anthropometric data such as weight and height and pregnant were excluded. Thus, data from 1746 subjects were used in this study (Fig. 1). Participants who did not undergo body composition measurements using BOD POD or DXA, but for whom other measures such as BMI and waist circumference were available, remained in the study because technical limitations of those tests such as the maximum weight supported by the equipment could exclude severely obese individuals (obesity class III).

The project corresponding to the fifth phase of the 1978/79 Ribeirao Preto birth cohort was approved by the Ethics Committee of the Ribeirao Preto Medical School (number 1.282.710). For this study, a new project was submitted to the Ethics Committee for ethical assessment under the terms of Resolution 466/12 of the National Health Council, requesting the waiver of a new informed consent form for the participants and authorization to use the information stored in the database through the term of commitment for data use. This study was approved under number 2.947.100. The names of the participants were not linked to the data to ensure confidentiality.

### Independent variables

The following independent variables that were available in the database of the fifth follow-up of the cohort were chosen based on previous studies: waist circumference (cm), body mass index (BMI) calculated by dividing weight (kg) by height in meters squared (m^2^), waist-to-height ratio (WHtR) (waist circumference divided by height), and fat mass percentage measured by BOD POD and DXA.

The anthropometric measures were obtained by a trained team according to standard techniques, with the subject barefoot and wearing light clothing. Weight was measured with a high-precision electronic scale to the nearest 100 g. Height was measured with a wall-mounted stadiometer to the nearest 0.1 cm. Waist circumference (WC) was measured at the midpoint between the last rib and the upper edge of the iliac crest with an inextensible measuring tape, with the subject standing and abdomen relaxed. The measurement was obtained at the end of a normal expiration.

Body fat percentage was obtained by BOD POD using the COSMED BodPod® Gold Standard, which calculates the body volume and density by measuring the air displaced in a closed chamber. Weight (kg) was also obtained with a high-precision scale coupled to the equipment. After weighing on the scale, two sequential measurements of body volume were obtained. The subject used tight fitting shorts and a sports bra for women, as well as a swim cap to compress the hair. In addition to weight and body volume, this equipment provides estimates of total fat mass and fat-free mass using pre-defined equations.

For the assessment of body fat percentage by DXA, a GE Healthcare® Lunar Prodigy DXA densitometer was used which evaluates body composition using the principle of X-ray attenuation by different body tissues, permitting the estimation of total lean mass, fat mass, and bone tissue. Each subject was subjected to a full body scan, lying completely still in the supine position on a table, with the legs together and arms along the body.

### Dependent variables

The outcome variables were FVC and FEV1. Spirometry was performed with a computer-controlled spirometer (Koko Digidoser System, PDS Instrumentation, Louisville, CO, USA), which was calibrated daily. The test was performed with the subject sitting and wearing a nose clip. The technical procedures, acceptability and reproducibility criteria adopted by the American Thoracic Society for spirometry were followed [28].

### Adjustment variables

**Fig. 2:**
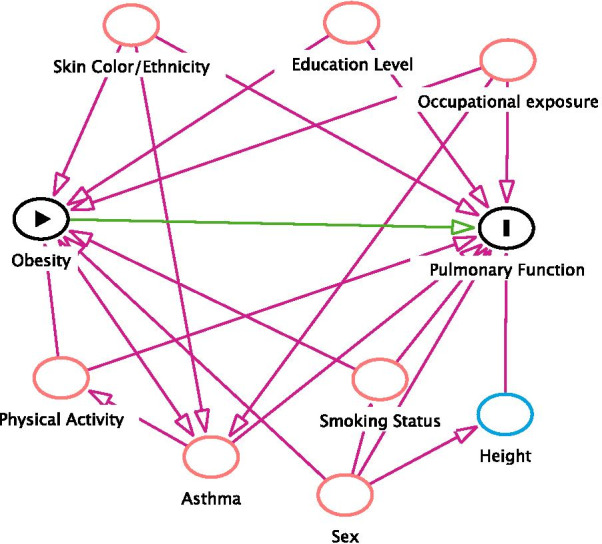
Legend. Directed acyclic graph (DAG) illustrating the causal effect of obesity on pulmonary function. Minimum adjustment to estimate the total effect of obesity on pulmonary function: skin color, education level, occupation, smoking, physical activity, sex, and asthma

A directed acyclic graph (DAG) was constructed using the DAGitty 2.3 software to verify the minimum adjustment necessary for the analysis model (Fig. 2). The DAG is a causal diagram built on already known theoretical assumptions regarding certain causal relationships [29]. Based on this diagram, the following variables were identified as potential confounders: self-reported skin color (white, black, brown, yellow, or other), educational level in years of schooling (illiterate, 1 to 4, 5 to 8, 9 to 11, and 12 or more years), occupation (first category: industry and construction, second category: less exposed, third category: no occupation), smoking (current, ex-smoker, never smoked), physical activity (classified as low, moderate and high according to the short form of the International Physical Activity Questionnaire—IPAQ [30]), and asthma (yes or no based on the following questions: Have you ever had asthma? and/or Did you experience wheezing in the last year?). Although the DAG identified sex as a confounding variable, we decided to stratify the analysis by sex instead of simply adjusting the model in order to permit comparison with other studies [18, 24].

### Statistical analysis

The statistical power of the sample was calculated, with a minimum number of 800 participants per group (men and women) and an R^2^ of 5% resulting in a power of 99% for this sample. For comparison of the characteristics between genders, the Student t-test was used for continuous variables and the chi-squared test for categorical variables. The association between body composition parameters and pulmonary function was evaluated using multiple linear regression models adjusted for the covariates identified by DAG. Adjustment for height, which is common in studies on obesity and pulmonary function, was not identified as a confounder in the DAG; however, height-adjusted analysis was also performed and the results are reported in supplemental material (Additional file 1: Table S1, S2).

For comparison of the models, the independent variables were standardized based on their means and standard deviations to analyze the associations with pulmonary function. The estimates, confidence intervals and R^2^ were thus used to contrast the models. The Stata v15 program was used for all analyses, adopting a level of significance of 5%. The following WHO classification of BMI categories was considered for description of the sample 1: low weight (BMI < 18.5 kg/m^2^), eutrophic (BMI ≥ 18.5 and < 25 kg/m^2^), overweight (BMI ≥ 25 and < 30 kg/m^2^), and obesity (BMI > 30 kg/m^2^).

## Results

The characteristics of the sample studied are shown in Table 1. The mean age was 38 years (38.12), with a slight predominance of female participants (52.3%). Most participants had a white skin color (79.1%) and more than 9 years of schooling (87.5%). The proportion of subjects working in industry and construction, who might be at higher risk of occupational exposure to hazardous materials, was low in men (7.1%) and women (5.1%). The prevalence of smokers did not differ between genders (*p* = 0.737). The presence of asthma was reported by 12.4% of men and 15.1% of women, with no significant difference (*p* = 0.084).

**Table 1:** Characteristics of the population studied (n = 1746). Ribeirão Preto, 2016/17

Variable	Male	Female	*p*-value
	n = 833 (47.7)	n = 913 (52.3)	
	n (%)	n (%)	
*Ethnicity*			0.0237
White	646 (77.6)	736 (80.6)	
Black	46 (5.5)	53 (5.8)	
Brown	137 (16.4)	122 (13.4)	
Yellow	4 (0.5)	2 (0.2)	
*Years of schooling*			0.330
Illiterate	–	1 (0.1)	
1–4	17 (2.0)	20 (2.2)	
5–8	89 (10.7)	87 (9.5)	
9–11	365 (43.8)	369 (40.5)	
≥ 12	360 (43.2)	435 (47.7)	
No information	2 (0.3)	–	
*Occupation*			0.001
Less exposed	734 (88.1)	784 (85.9)	
Industry/construction	59 (7.1)	47 (5.1)	
No occupation	40 (4.8)	82 (9.0)	
*Smoking*			0.737
Never	595 (71.4)	648 (71.0)	
Ex-smoker	112 (13.5)	116 (12.7)	
Current smoker	124 (14.9)	147 (16.1)	
No information	2 (0.2)	2 (0.2)	
*Asthma*			0.084
Yes	103 (12.4)	139 (15.2)	
No	730 (87.6)	774 (84.8)	
*Physical activity*			0.185
Low	386 (46.3)	391 (42.8)	
Moderate	312 (37.5)	381 (41.7)	
High	135 (16.2)	141 (15.5)	
*BMI*			< 0.001
Low weight (< 18.5)	5 (0.6)	9 (1)	
Eutrophic (≥ 18.5 < 25)	159 (19.1)	281 (30.8)	
Overweight (≥ 25 < 30)	371 (44.5)	315 (34.5)	
Obesity (≥ 30)	298 (35.8)	308 (33.7)	

Asthma was defined by the questions: Have you ever had asthma? Did you experience wheezing in the last year? Physical activity level was assessed by the International Physical Activity Questionnaire. BMI: body mass index

As can be seen in Table 2, mean height, weight, waist circumference and WHtR were higher in men. The body composition measurements showed a higher fat mass percentage in women. There was a higher proportion of overweight men (44.5%) than women (34.5%) (*p* < 0.001). The mean spirometric values (in absolute numbers, L or L/s) were higher in men, as expected (*p* < 0.001) (Table 2).

**Table 2:** Description of anthropometric, body composition and spirometric values of the population studied (n = 1746). Riberão Preto, 2016/17

Variable	Male	Female	*p*-value
	n = 833 (47.7%)	n = 913 (52.3%)	
	Mean (SD)	Mean (SD)	
Height (cm)	175.3 (6.62)	162.5 (6.27)	< 0.001
Weight (kg)	89.13 (16.89)	75.37 (16.87)	< 0.001
BMI	28.94 (5.15)	28.54 (6.17)	0.14
Waist circumference (cm)	97.96 (12.52)	87.97 (13.23)	< 0.001
Waist-height ratio	0.55 (0.07)	0.54 (0.08)	< 0.001
BOD POD-measured fat mass (%)	25.95 (8.47)	38.31 (8.71)	< 0.001
DXA-measured fat mass (%)	28.68 (7.86)	41.45 (7.37)	< 0.001
FEV1 (L)	3.83 (0.58)	2.84 (0.40)	< 0.001
FVC (L)	4.89 (0.72)	3.53 (0.53)	< 0.001
PEF (L/s)	9.6 (1.80)	6.7 (1.07)	< 0.001
FEF 25–75% (L/s)	3.61 (1.04)	2.89 (0.76)	< 0.001

BMI: body mass index

Unadjusted linear regression analysis revealed a negative association of BMI, WHtR and BOD POD- and DXA-measured fat mass percentage with FEV1 in men and women. FEV1 was not associated with waist circumference in men, it was in women (Tables 3 and 4). For FVC, all parameters showed a negative association in men and women, except for waist circumference.

**Table 3:** Linear regression analysis in men

	Unadjusted model		*p*-value	R^2^	Adjusted model*		*p*-value	R^2^
	β	95% CI			β	95% CI		
*FEV1*								
BMI SD	− 0.071	(− 0.115, − 0.028)	0.001	0.012	− 0.060	(− 0.103, − 0.018)	0.005	0.088
WC SD	− 0.041	(− 0.084, 0.003)	0.067	0.004	− 0.027	(− 0.070, 0.016)	0.215	0.081
WHtR SD	− 0.126	(− 0.169, − 0.084)	< 0.001	0.039	− 0.109	(− 0.151, − 0.067)	< 0.001	0.107
FM-BODPOD SD	− 0.137	(− 0.186, − 0.087)	< 0.001	0.036	− 0.134	(− 0.183, − 0.085)	< 0.001	0.110
FM-DXA SD	− 0.104	(− 0.158, − 0.049)	< 0.001	0.021	− 0.113	(− 0.167, − 0.059)	< 0.001	0.103
*FVC*								
BMI SD	− 0.085	(− 0.139, − 0.031)	0.002	0.011	− 0.074	(− 0.127, − 0.020)	0.007	0.058
WC SD	− 0.024	(− 0.079, 0.030)	0.380	0.001	− 0.011	(− 0.066, 0.043)	0.678	0.050
WHtR SD	− 0.151	(− 0.204, − 0.098)	< 0.001	0.036	− 0.133	(− 0.187, − 0.080)	< 0.001	0.077
FM-BODPOD SD	− 0.169	(− 0.231, − 0.107)	< 0.001	0.035	− 0.174	(− 0.236, − 0.112)	< 0.001	0.085
FM-DXA SD	− 0.095	(− 0.163, − 0.028)	0.006	0.021	− 0.118	(− 0.187, − 0.050)	< 0.001	0.058

^*^Model adjusted for skin color, education level, occupation, smoking, physical activity, and asthma. 95% CI: 95% confidence interval; FEV1: forced expiratory volume in 1 s; FVC: forced vital capacity; SD: standard deviation; BMI: body mass index; WC: waist circumference; WHtR: waist-height ratio; FM-BODPOD: fat mass measured by BOD POD; FM-DXA: fat mass measured by DXA

**Table 4:** Linear regression analysis in women

	Unadjusted model		*p*-value	R^2^	Adjusted model*		*p*-value	R^2^
	β	95% CI			β	95% CI		
*FEV1*								
BMI SD	− 0.046	(− 0.071, − 0.022)	< 0.001	0.015	− 0.030	(− 0.054, − 0.006)	0.014	0.073
WC SD	− 0.038	(− 0.065, − 0.011)	0.007	0.008	− 0.017	(− 0.045, 0.010)	0.220	0.068
WHtR SD	− 0.083	(− 0.107, − 0.059)	< 0.001	0.047	− 0.064	(− 0.088, − 0.039)	< 0.001	0.092
FM-BODPOD SD	− 0.089	(− 0.121, − 0.057)	< 0.001	0.032	− 0.071	(− 0.103, − 0.039)	< 0.001	0.086
FM-DXA SD	− 0.076	(− 0.113, − 0.039)	< 0.001	0.020	− 0.061	(− 0.099, − 0.024)	0.001	0.071
*FVC*								
BMI SD	− 0.053	(− 0.085, − 0.021)	0.001	0.011	− 0.035	(− 0.067, − 0.003)	0.033	0.054
WC SD	− 0.029	(− 0.065, 0.008)	0.120	0.003	− 0.005	(− 0.042, 0.032)	0.783	0.049
WHtR SD	− 0.098	(− 0.130, − 0.066)	< 0.001	0.038	− 0.077	(− 0.110, − 0.044)	< 0.001	0.071
FM-BODPOD SD	− 0.107	(− 0.150, − 0.065)	< 0.001	0.027	− 0.086	(− 0.129, − 0.043)	< 0.001	0.064
FM-DXA SD	− 0.097	(− 0.146, − 0.048)	< 0.001	0.020	− 0.076	(− 0.126, − 0.026)	0.003	0.059

^*^Model adjusted for skin color, education level, occupation, smoking, physical activity, and asthma. 95% CI: 95% confidence interval; FEV1: forced expiratory volume in 1 s; FVC: forced vital capacity; SD: standard deviation; BMI: body mass index; WC: waist circumference; WHtR: waist–height ratio; FM-BODPOD: fat mass measured by BOD POD; FM-DXA: fat mass measured by DXA

After adjusting the analysis for confounders, most body composition variables (BMI, WHtR and BOD POD- and DXA-measured fat mass percentage) were negatively associated with FEV1 and FVC in men and women. Only waist circumference was not associated with either FEV1 or FVC.

Considering the model adjusted for skin color, education level, occupation, smoking, physical activity, and asthma in men and women, and observing the coefficients of association, body fat measured by the BOD POD method was associated with greater negative variation than the other methods, especially in men. The observation of confidence intervals confirmed these findings that are attenuated in women.

### Supplemental results

The analysis was also adjusted for height, added to all the adjusting variables described above. Results are very similar, except for the fact that waist circumference is now associated to FEV1 and FVC reduction. This means that waist circumference is a good obesity parameter if controlled by height. Even though, it is not as good as the other variables, as shown by the negative association values that are lower than those relative to fat mass measurements (Additional file 1).

## Discussion

This study found a negative association of anthropometric (BMI and WHtR) and body composition parameters (DXA- and BOD POD-measured fat mass) with pulmonary function assessed by FEV1 and FVC in both genders, particularly in men. The adjusted models showed that, for every increase of 1 kg/m^2^ in BMI, FVC decreased by 12 ml in men and by 6 ml in women and FEV1 decreased by 10 ml and 5 ml, respectively. Regarding the body composition parameters, for every 1% increase in BOD POD-measured fat mass, FVC decreased by 15 ml in men and by 8 ml in women and FEV1 decreased by 12 ml and 6 ml, respectively. Similar values were obtained for the DXA measurements in which FVC decreased by 11 ml in men and by 7 ml in women for every 1% increase in fat mass and FEV1 decreased by 11 ml and 6 ml, respectively (Additional file 1: Table S3).

Rowe et al. [31] evaluated the association of different anthropometric measures (BMI, waist circumference, hip circumference, waist–hip ratio, WHtR, and skinfolds) with pulmonary function assessed by FVC and FEV1. Although the authors did not report height as a confounding variable in the methods, the linear regression models were adjusted for height, except for the models using BMI and WHtR. The R^2^ was used as a measure to compare the associations. The associations with the highest R^2^ for both FEV1 and FVC in men and women were obtained when skinfold thickness was used, followed by waist circumference, and the worst associations were obtained using BMI and WHtR. These results suggest that height is an important factor in the study of associations and that it can be addressed in different ways.

Some previous studies have shown that waist circumference is a better marker of obesity than BMI in the association with pulmonary function. This can be explained by the fact that waist circumference is a direct marker of central obesity and an indirect marker of visceral fat accumulation; however, those studies adjusted for height in their analyses [2, 6, 32, 33]. In the present study, when height was added to the model (supplemental results), waist circumference was found to be significantly associated with FEV1 in men and women and with FVC in men. Each 1-cm increase in waist circumference decreased FEV1 and FVC by 6 ml in men and by 2 ml in women.

An increasing number of studies have demonstrated the importance of assessing obesity beyond BMI, using more accurate measures of the amount and distribution of body fat, and differentiating fat mass from lean mass. Methods such as bioimpedance, computed tomography, magnetic resonance imaging, BOD POD, and DXA have been employed in an attempt to avoid erroneous classifications of obesity based only on BMI [34]. Today, DXA is considered the gold standard for this assessment [35, 36].

In all models, BOD POD-measured body fat was associated with a greater negative variation in both FEV1 and FVC in men and women when compared to the other methods. Similar values were observed for the association of pulmonary function with DXA-measured fat mass, indicating that the relationship of obesity with pulmonary function is based on the content and endocrinological function of fat mass, in addition to the respiratory mechanical injury. Recent studies using fat mass measured by computed tomography found that both visceral adipose tissue and total adipose tissue are associated with poor pulmonary function, irrespective of waist circumference [22, 36, 37].

Obesity is known to affect pulmonary function regardless of the presence of respiratory, cardiovascular, or metabolic diseases [4, 7, 8], interfering with respiratory mechanics and lung-thorax compliance. Furthermore, studies have demonstrated that adipose tissue, especially the visceral adipose tissue, is an active tissue in terms of inflammation (cytokine production) and endocrinological activity, releasing hormones that also interfere with pulmonary function [13, 15, 16].

This study has several strengths. It has used cross-sectional data from a birth cohort, which allowed us to obtain a large and representative sample of the population studied, in addition to being subjected to standardization and quality techniques of a longitudinal study in which the responsibility to record detailed quality data is fundamental. Additionally, the study employed sophisticated and accurate methods for body composition measurement. On the other hand, the study has limitations. Its cross-sectional design does not allow to evaluate the causal relationship between body composition and pulmonary function reduction, nor how weight evolution over the time influences the maximal lung function and its decline over the years. The study used a population sample with a limited age range from the city of Ribeirão Preto, whose socioeconomic pattern differs from that of most other Brazilian cities, impairing generalization of our findings to other ages and regions. The original project also did not have this study as the central objective. It is difficult to extract data from a questionnaire that was predefined with other initial general objectives. Some anthropometric parameters such as hip circumference and skinfold thickness that could be useful for comparison with other publications were not measured.

There are other variables that can confuse the association between body composition and pulmonary function, such as diet and socioeconomic status which were not included. Another limitation is that pulmonary function was evaluated only by spirometry; static volumes measurements would certainly improve this study. It is known that obesity can change other functional parameters such as residual volume and gas exchange variables, which spirometry does not measure.

Since reduced pulmonary function and obesity have been consistently shown to be independent predictors of morbidity and mortality and previous studies have demonstrated an association between the two, identification of the method that best demonstrates this association can provide more information about the interaction between adiposity and lung physiology. The present data demonstrate the advantage of more accurate measurements of fat content (BOD POD and DXA) over anthropometric measures. The results mainly indicate the inferiority of the one-dimensional variable waist circumference, which failed to demonstrate the association that was evidenced by WHtR and BMI, probably because waist circumference is a variable that does not contain height. This understanding may be important for future studies, for clinical assessments, and for elaboration of public health policies.

## Conclusion

Anthropometric measures of excess weight and increased body composition parameters were associated with lower FVC and FEV1. These associations were more expressive in men. Furthermore, in the study of the association between obesity and pulmonary function, measures that include height for their calculation, such as WHtR, are sufficient and somehow equivalent to more sophisticated body composition parameters obtained by BOD POD and DXA.

## Supplementary Information

**Additional file 1: Table S1.** Title. Linear regression models adjusted for confounder variables and for height in men. Legend. *Model 1 adjusted for skin color, education level, occupation, smoking, physical activity, and asthma. **Model 2: Model 1 + height. 95% CI: 95% confidence interval; FEV1: forced expiratory volume in 1 s; FVC: forced vital capacity; SD: standard deviation; BMI: body mass index; WC: waist circumference; WHtR: waist–height ratio; FM-BODPOD: fat mass measured by BOD POD; FM-DXA: fat mass measured by DXA. **Table S2.** Title. Linear regression models adjusted for confounder variables and for height in women. Legend. *Model 1 adjusted for skin color, education level, occupation, smoking, physical activity, and asthma. **Model 2: Model 1 + height. 95% CI: 95% confidence interval; FEV1: forced expiratory volume in 1 s; FVC: forced vital capacity; SD: standard deviation; BMI: body mass index; WC: waist circumference; WHtR: waist–height ratio; FM-BODPOD: fat mass measured by BOD POD; FM-DXA: fat mass measured by DXA. **Table S3.** Linear regression adjusted for unstandardized independent variables. Legend. Model adjusted for skin color, education level, occupation, smoking, physical activity, and asthma. 95% CI: 95% confidence interval; LL: lower limit; UL: upper limit; FEV1: forced expiratory volume in 1 s; FVC: forced vital capacity; BMI: body mass index; WC: waist circumference; WHtR: waist-height ratio; FM-BODPOD: fat mass measured by BOD POD; FM-DXA: fat mass measured by DXA.

## Data Availability

The datasets used and/or analyzed during the current study are available from the corresponding author on reasonable request.

## Abbreviations

BMI: Body mass index
BOD POD: Air-displacement plethysmography
cm: Centimeters
DAG: Diggity or directed acyclic graphic
DXA: Dual-energy X-ray absorptiometry
FEV1: Forced expiratory volume in 1 s
FVC: Forced vital capacity
g: Grams
IPAQ: International Physical Activity Questionnaire
kg: Kilograms
m: Meters
ml: Millimeters
SP: São Paulo
WHtR: Waist–height ratio
WHO: World Health Organization
