# Supplementary material for: Comparison of body composition parameters in the study of the association between body composition and pulmonary function
Source: BMC Pulm Med. 2021 May 25;21:178. doi: 10.1186/s12890-021-01543-1 (PMC8146631; doi:10.1186/s12890-021-01543-1)
Supplement: Supplementary file 1 — Additional file 1: Table S1. Title. Linear regression models adjusted for confounder variables and for height in men. Legend. *Model 1 adjusted for skin color, education level, occupation, smoking, physical activity, and asthma. **Model 2: Model 1 + height. 95% CI: 95% confidence interval; FEV1: forced expiratory volume in 1 s; FVC: forced vital capacity; SD: standard deviation; BMI: body mass index; WC: waist circumference; WHtR: waist–height ratio; FM-BODPOD: fat mass measured by BOD POD; FM-DXA: fat mass measured by DXA. Table S2. Title. Linear regression models adjusted for confounder variables and for height in women. Legend. *Model 1 adjusted for skin color, education level, occupation, smoking, physical activity, and asthma. **Model 2: Model 1 + height. 95% CI: 95% confidence interval; FEV1: forced expiratory volume in 1 s; FVC: forced vital capacity; SD: standard deviation; BMI: body mass index; WC: waist circumference; WHtR: waist–height ratio; FM-BODPOD: fat mass measured by BOD POD; FM-DXA: fat mass measured by DXA. Table S3. Linear regression adjusted for unstandardized independent variables. Legend. Model adjusted for skin color, education level, occupation, smoking, physical activity, and asthma. 95% CI: 95% confidence interval; LL: lower limit; UL: upper limit; FEV1: forced expiratory volume in 1 s; FVC: forced vital capacity; BMI: body mass index; WC: waist circumference; WHtR: waist-height ratio; FM-BODPOD: fat mass measured by BOD POD; FM-DXA: fat mass measured by DXA. [file 12890_2021_1543_MOESM1_ESM.docx]

Table S1

|  |  | Unadjusted model  β                  95% CI | | p-value | Adjusted model 1*  β                95% CI | | p-value | Adjusted model 2**  β                 95% CI | | p-value |
| --- | --- | --- | --- | --- | --- | --- | --- | --- | --- | --- |
|  | BMI SD | -0.071 | (-0.115, -0.028) | 0.001 | -0.060 | (-0.103, -0.018) | 0.005 | -0.062 | (-0.099, -0.024) | 0.001 |
|  | WC SD | -0.041 | (-0.084, 0.003) | 0.067 | -0.027 | (-0.070, 0.016) | 0.215 | -0.078 | (-0.116, -0.039) | <0.001 |
| FEV1 | WHtR SD | -0.126 | (-0.169, -0.084) | <0.001 | -0.109 | (-0.151, -0.067) | <0.001 | -0.076 | (-0.114, -0.038) | <0.001 |
|  | FM - BODPOD SD | -0.137 | (-0.186, -0.087) | <0.001 | -0.134 | (-0.183, -0.085) | <0.001 | -0,141 | (-0.184, -0.098) | <0.001 |
|  | FM - DXA SD | -0.104 | (-0.158, -0.049) | <0.001 | -0.113 | (-0.167, - 0.059) | <0.001 | -0,131 | (-0.178, -0.083) | <0.001 |
|  |  |  |  |  |  |  |  |  |  |  |
|  | BMI SD | -0.085 | (-0.139, -0.031) | 0.002 | -0.074 | (-0.127, -0.020) | 0.007 | -0,076 | (-0.121, -0.030) | 0.001 |
|  | WC SD | -0.024 | (-0.079, 0.030) | 0.380 | -0.011 | (-0.066, 0.043) | 0.678 | -0,087 | (-0.133, -0.041) | <0.001 |
| FVC | WHtR SD | -0.151 | (-0.204, -0.098) | <0.001 | -0.133 | (-0.187, -0.080) | <0.001 | -0,085 | (-0.130, -0.039) | <0.001 |
|  | FM - BODPOD SD | -0.169 | (-0.231, -0.107) | <0.001 | -0.174 | (-0.236, -0.112) | <0.001 | -0,184 | (-0.236, -0.132) | <0.001 |
|  | FM - DXA SD | -0.095 | (-0.163, -0.028) | 0.006 | -0.118 | (-0.187, -0.050) | <0.001 | -0,144 | (-0.201, -0.087) | <0.001 |

Table S2

|  |  | Unadjusted model  β          95% CI | | p-value | Adjusted model 1*  β       95% CI | | p-value | Adjusted model 2*  β        95% CI | | p-value |
| --- | --- | --- | --- | --- | --- | --- | --- | --- | --- | --- |
|  | BMI SD | -0.046 | (-0.071, -0.022) | <0.001 | -0.030 | (-0.054, -0.006) | 0.014 | -0.021 | (-0.042, 0.000) | 0.049 |
|  | WC SD | -0.038 | (-0.065, -0.011) | 0.007 | -0.017 | (-0.045, 0.010) | 0.220 | -0.031 | (-0.055, -0.007) | 0.012 |
| FEV1 | WHtR SD | -0.083 | (-0.107, -0.059) | <0.001 | -0.064 | (-0.088, -0.039) | <0.001 | -0.030 | (-0.052, -0.008) | 0.009 |
|  | FM - BODPOD SD | -0.089 | (-0.121, -0.057) | <0.001 | -0.071 | (-0.103, -0.039) | <0.001 | -0.068 | (-0.096, -0.039) | <0.001 |
|  | FM - DXA SD | -0.076 | (-0.113, -0.039) | <0.001 | -0.061 | (-0.099, -0.024) | 0.001 | -0.055 | (-0.087, -0.022) | 0.001 |
|  |  |  |  |  |  |  |  |  |  |  |
|  | BMI SD | -0.053 | (-0.085, -0.021) | 0.001 | -0.035 | (-0.067, -0.003) | 0.033 | -0.021 | (-0.048, 0.006) | 0.121 |
|  | WC SD | -0.029 | (-0.065, 0.008) | 0.120 | -0.005 | (-0.042, 0.032) | 0.783 | -0.026 | (-0.056, 0.005) | 0.101 |
| FVC | WHtR SD | -0.098 | (-0.130, -0.066) | <0.001 | -0.077 | (-0.110, -0.044) | <0.001 | -0.025 | (-0.054, 0.003) | 0.079 |
|  | FM - BODPOD SD | -0.107 | (-0.150, -0.065) | <0.001 | -0.086 | (-0.129, -0.043) | <0.001 | -0.081 | (-0.117, -0.045) | <0.001 |
|  | FM - DXA SD | -0.097 | (-0.146, -0.048) | <0.001 | -0.076 | (-0.126, -0.026) | 0.003 | -0.066 | (-0.108, -0.025) | 0.002 |

Table S3

|  |  | Male | | | | |  | Female | | | | |
| --- | --- | --- | --- | --- | --- | --- | --- | --- | --- | --- | --- | --- |
|  |  |  |  | 95% CI | |  |  |  |  | 95% CI | |  |
| Dependent variable | Independent variable | Estimate | p-value | LL | UL | R^2^ |  | Estimate | p-value | LL | UL | R^2^ |
|  | BMI | -0.010 | <0.01 | -0.017 | -0.003 | 0.087 |  | -0.005 | 0.014 | -0.009 | -0.001 | 0.072 |
|  | WC | -0.001 | 0.21 | -0.005 | 0.001 | 0.080 |  | -0.001 | 0.22 | -0.003 | 0.000 | 0.067 |
| FEV1 | WHtR | -1.379 | <0.01 | -1.910 | -0.846 | 0.107 |  | -0.809 | <0.01 | -1.121 | -0.497 | 0.092 |
|  | FM - BODPOD | -0.012 | <0.01 | -0.017 | -0.008 | 0.110 |  | -0.006 | <0.01 | -0.009 | -0.003 | 0.086 |
|  | FM - DXA | -0.011 | <0.01 | -0.016 | -0.005 | 0.103 |  | -0.006 | <0.01 | -0.009 | -0.002 | 0.071 |
|  |  |  |  |  |  |  |  |  |  |  |  |  |
|  | BMI | -0.012 | <0.01 | -0.022 | -0.003 | 0.058 |  | -0.006 | 0.03 | -0.011 | -0.000 | 0.053 |
|  | WC | -0.000 | 0.67 | -0.004 | 0.003 | 0.050 |  | -0.000 | 0.78 | -0.003 | 0.002 | 0.049 |
| FVC | WHtR | -1.695 | <0.01 | -2.372 | -1.017 | 0.077 |  | -0.982 | <0.01 | -1.401 | -0.564 | 0.071 |
|  | FM - BODPOD | -0.015 | <0.01 | -0.022 | -0.010 | 0.084 |  | -0.008 | <0.01 | -0.012 | -0.004 | 0.064 |
|  | FM - DXA | -0.011 | <0.01 | -0.018 | -0.005 | 0.058 |  | -0.007 | <0.01 | -0.012 | -0.002 | 0.059 |
